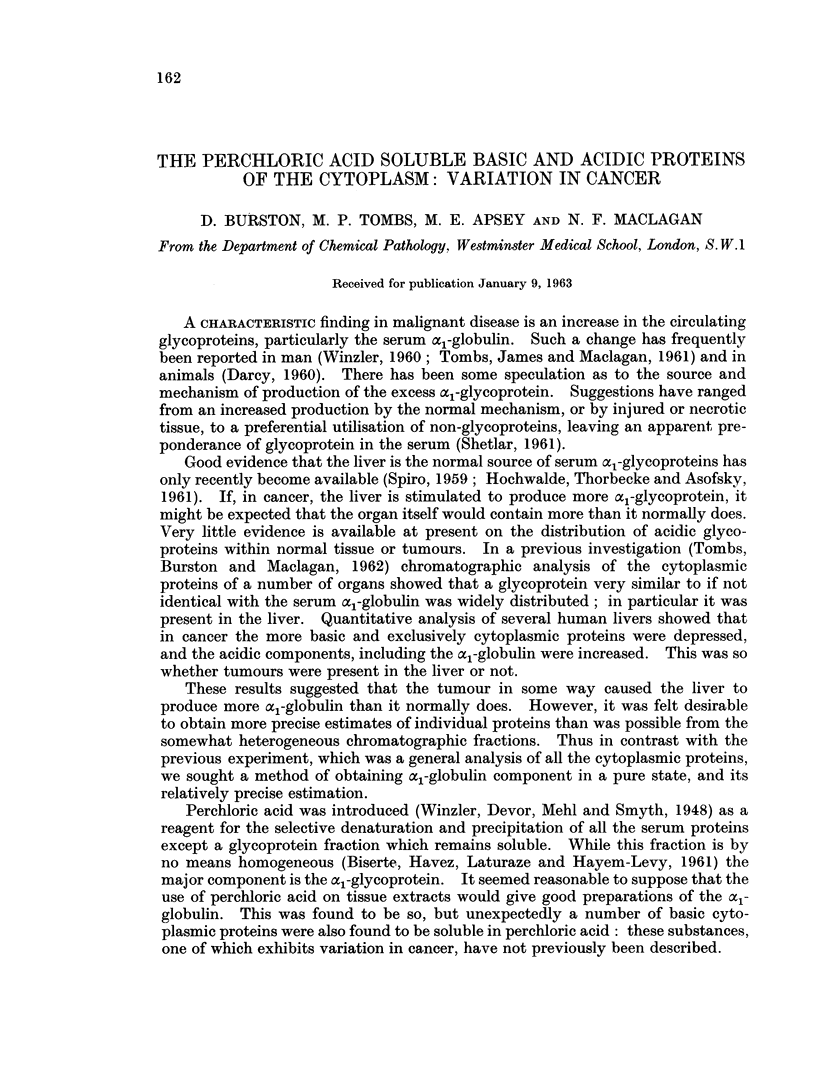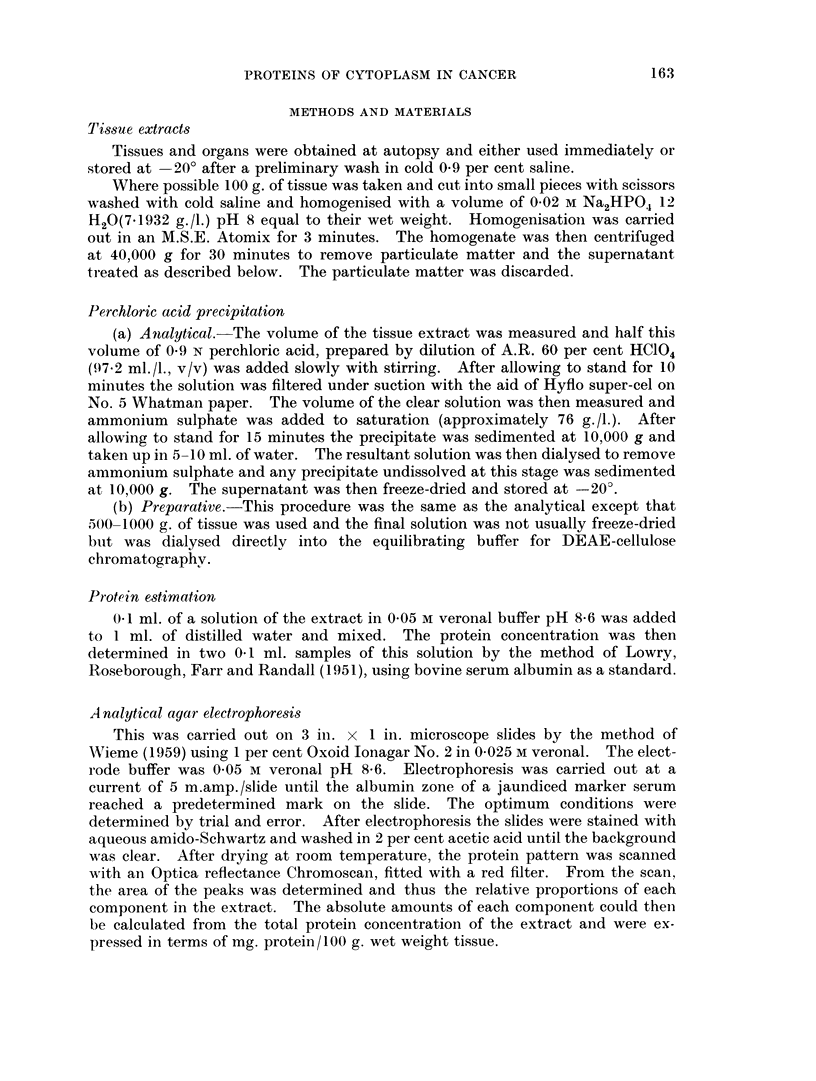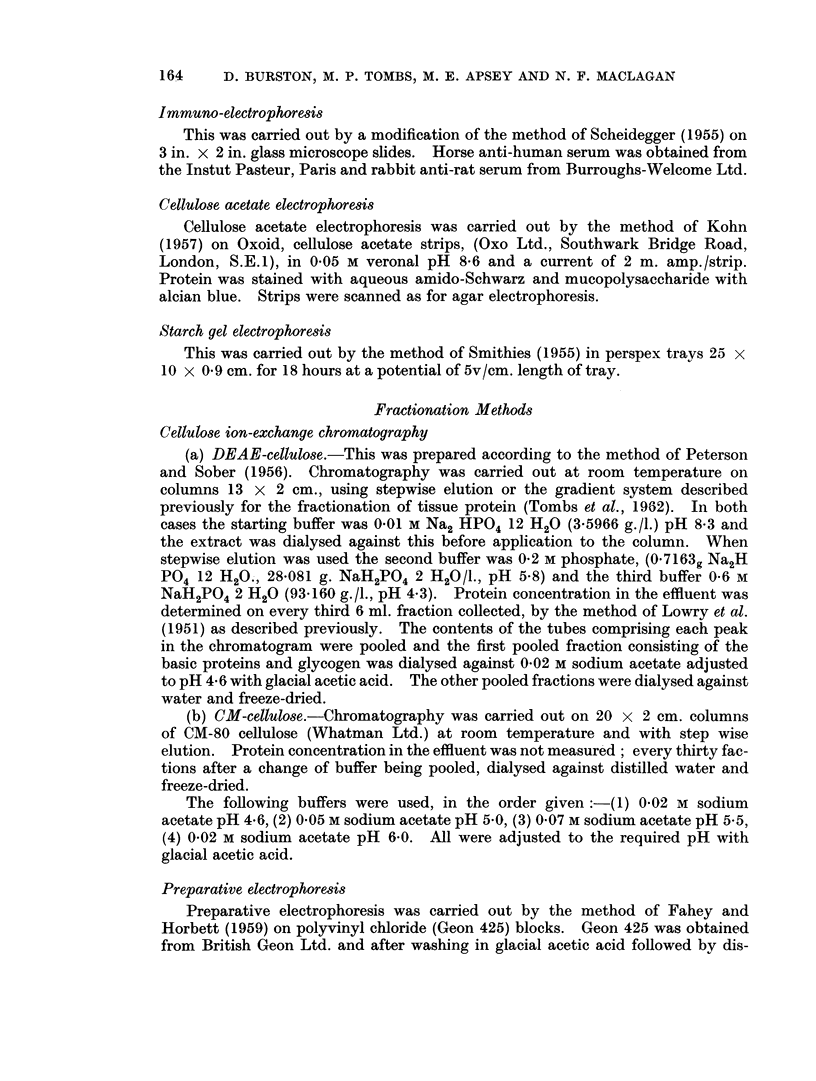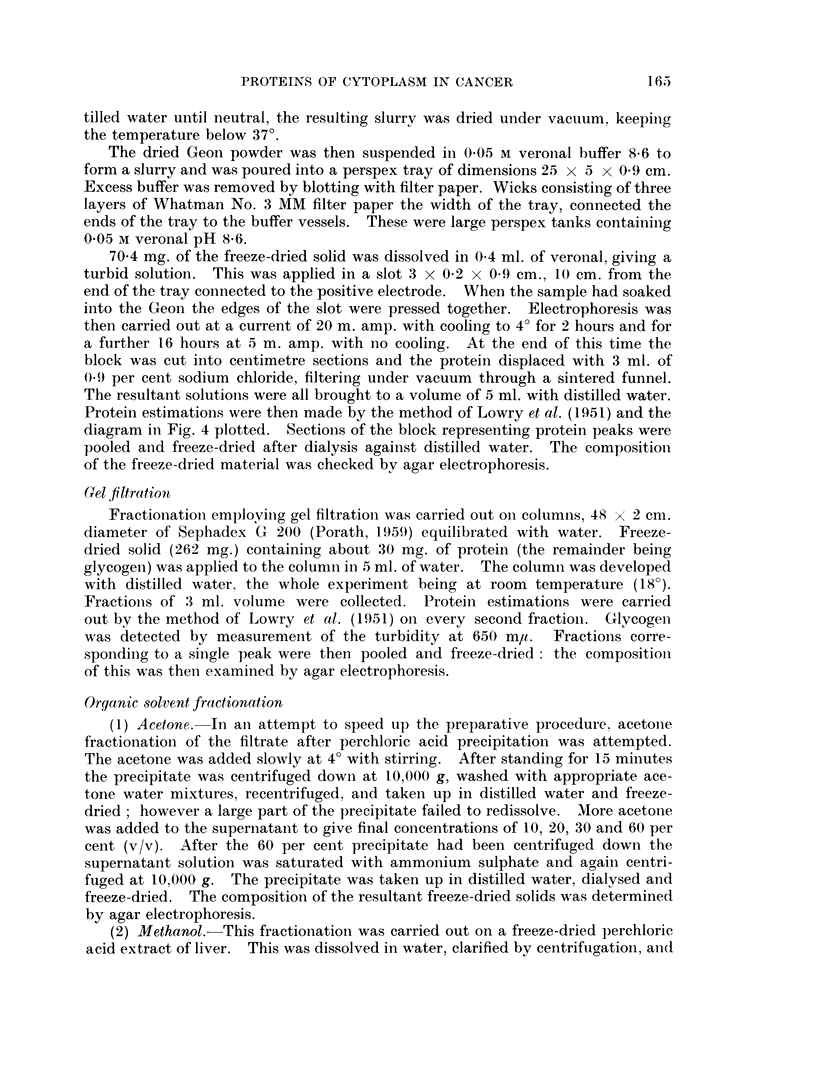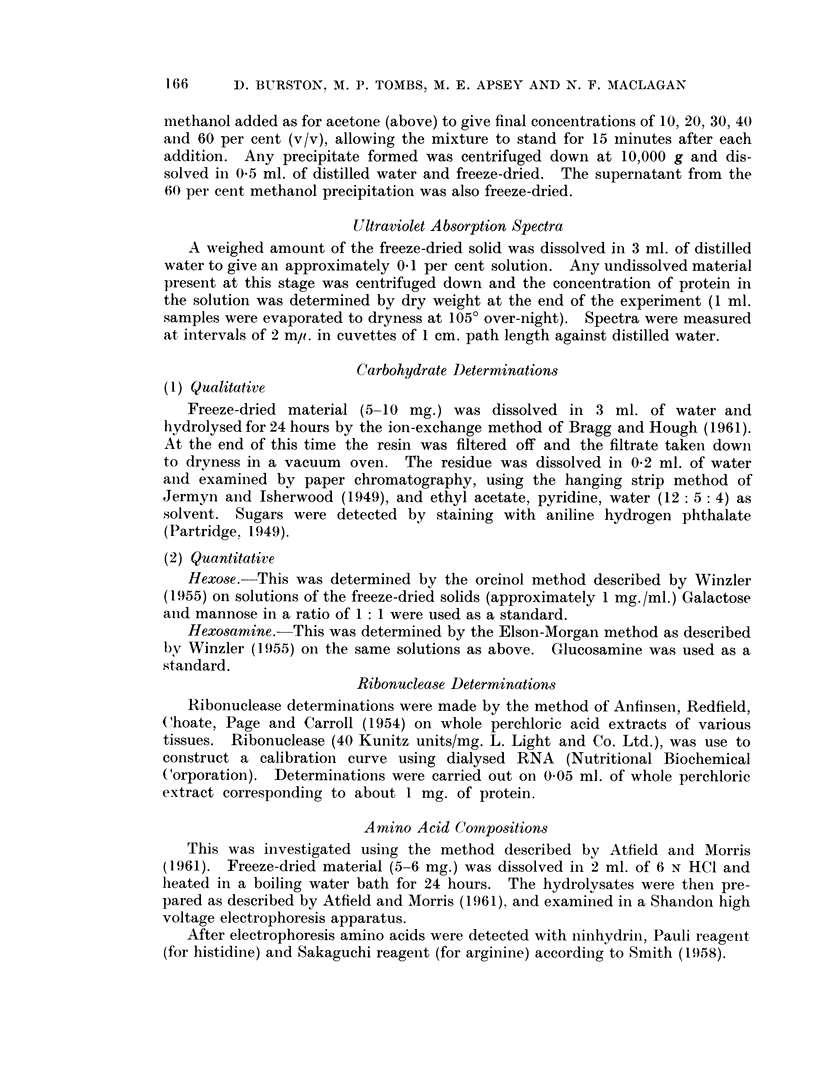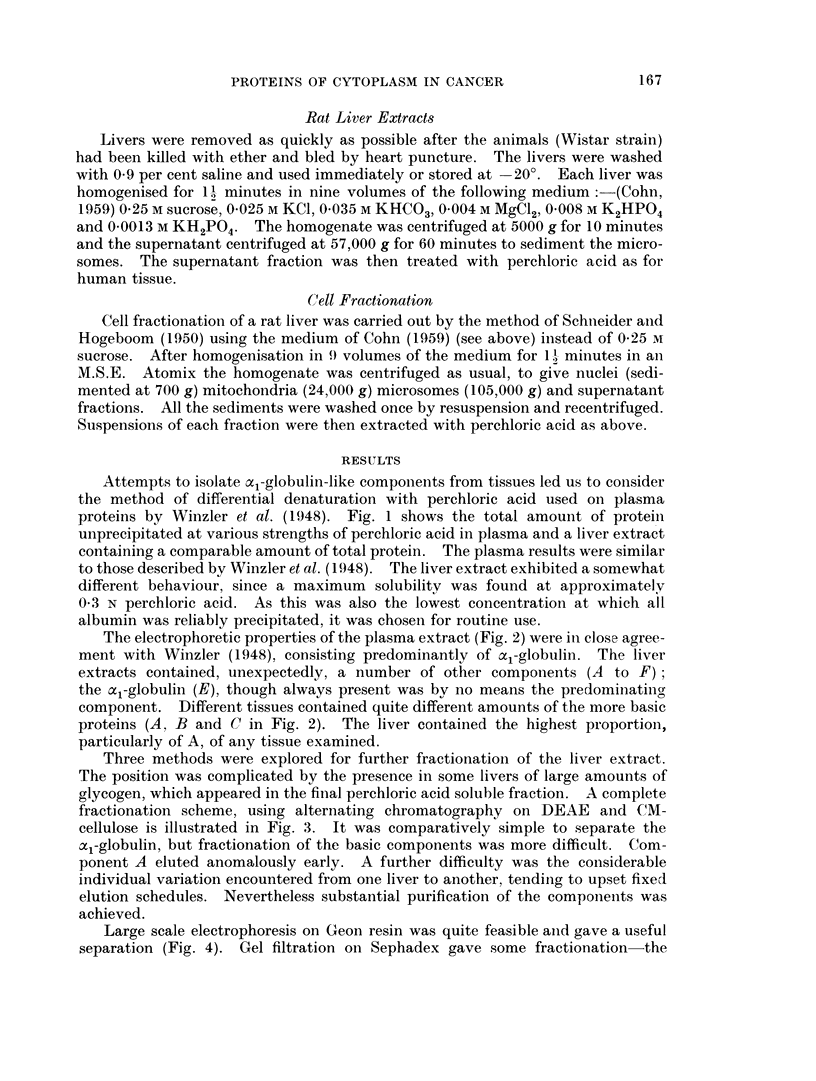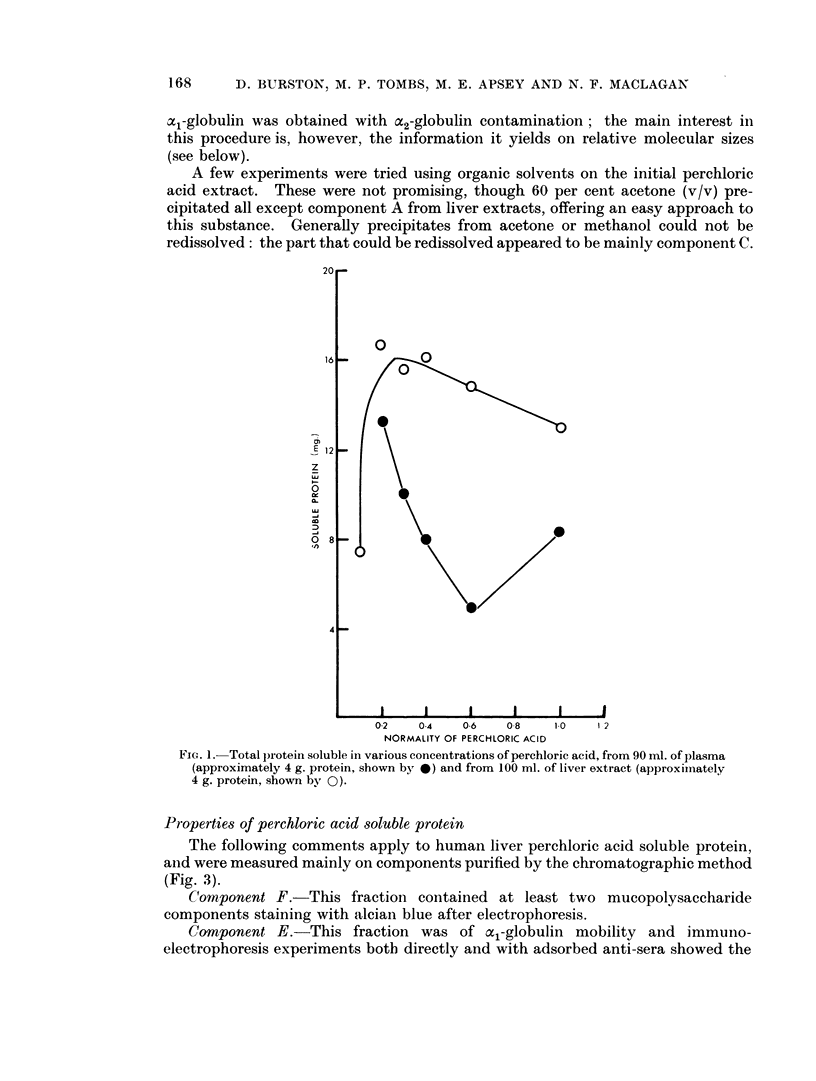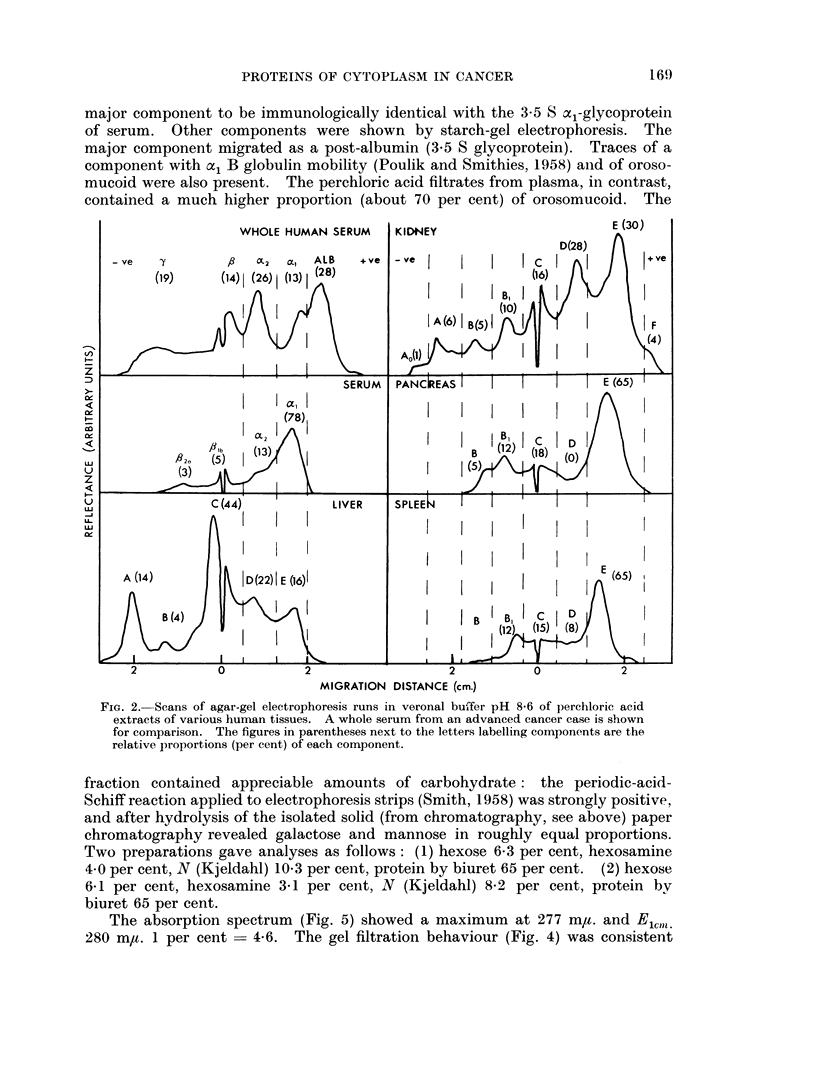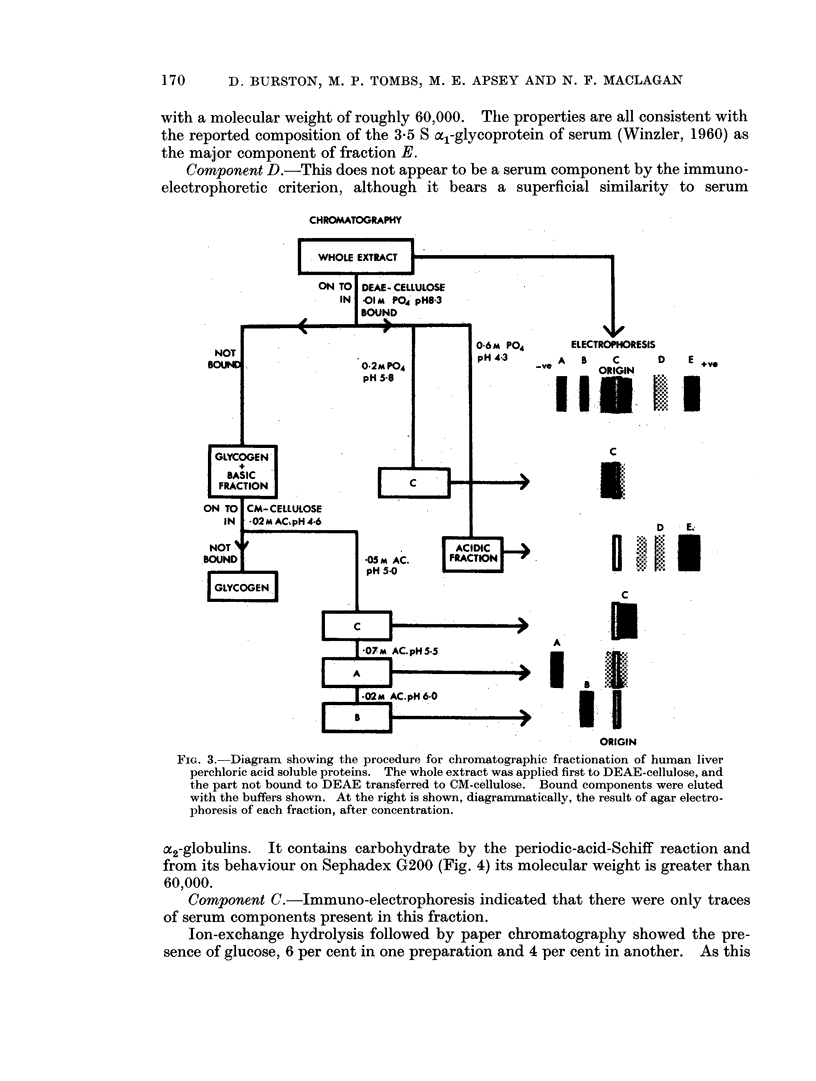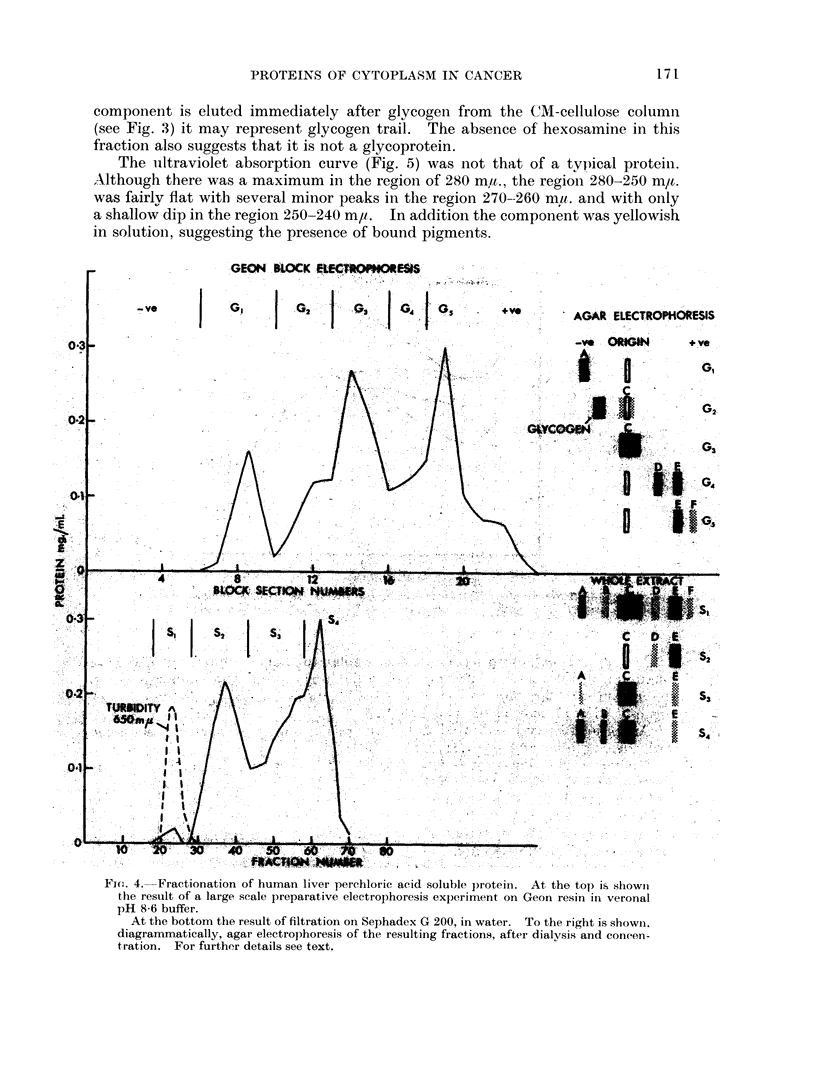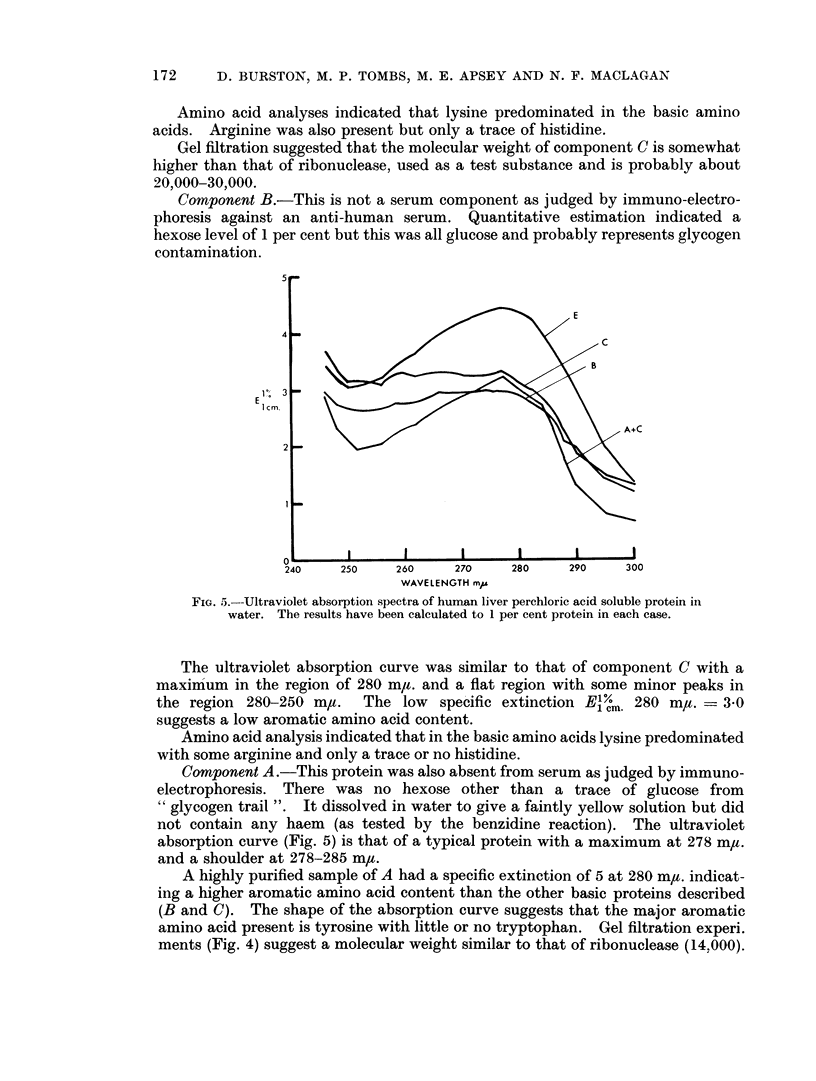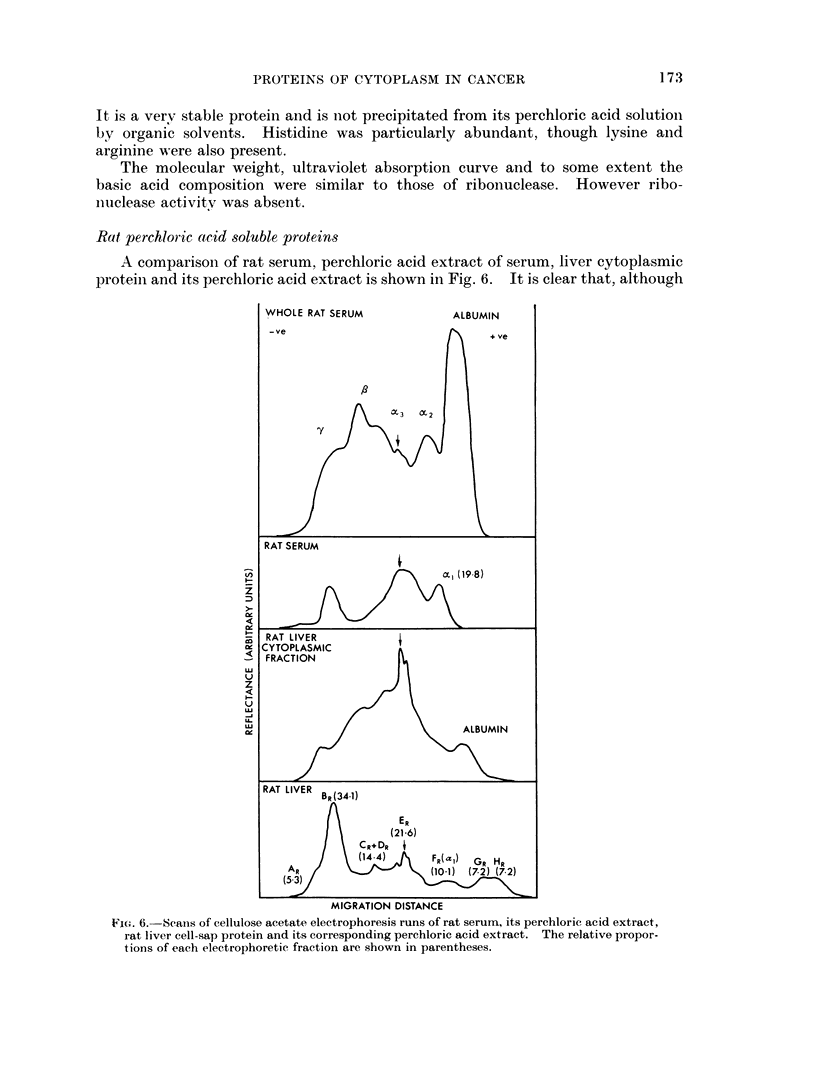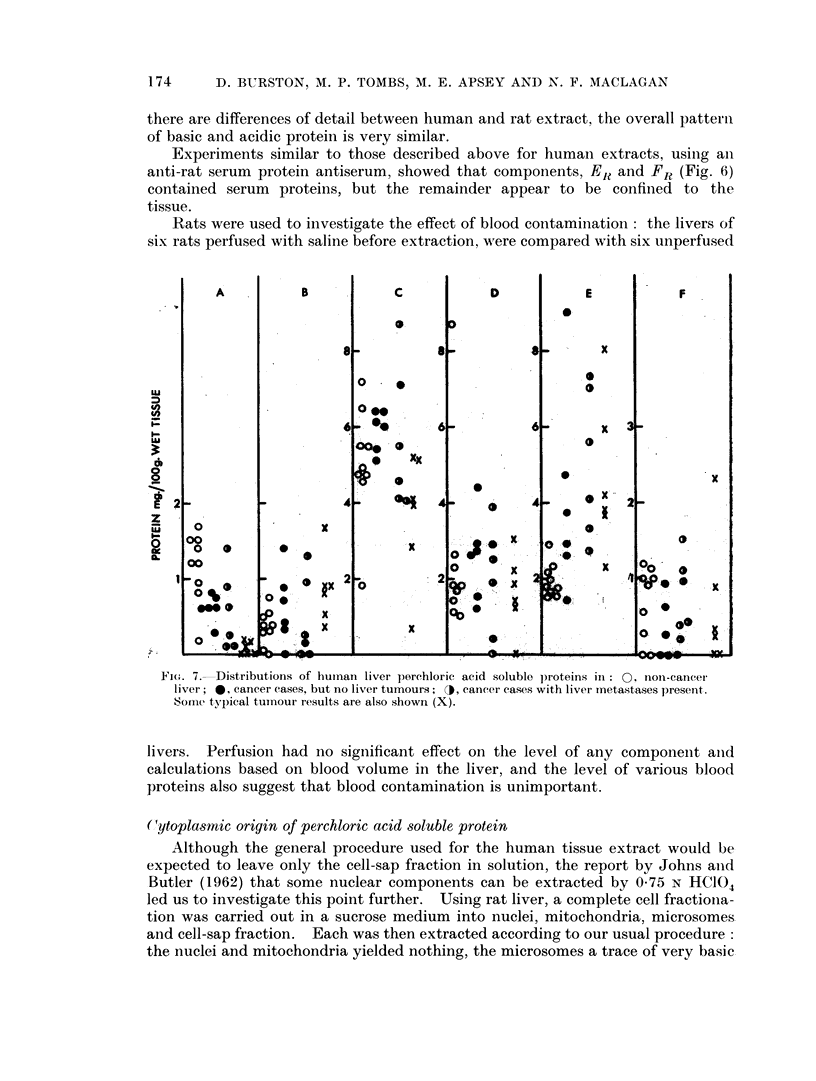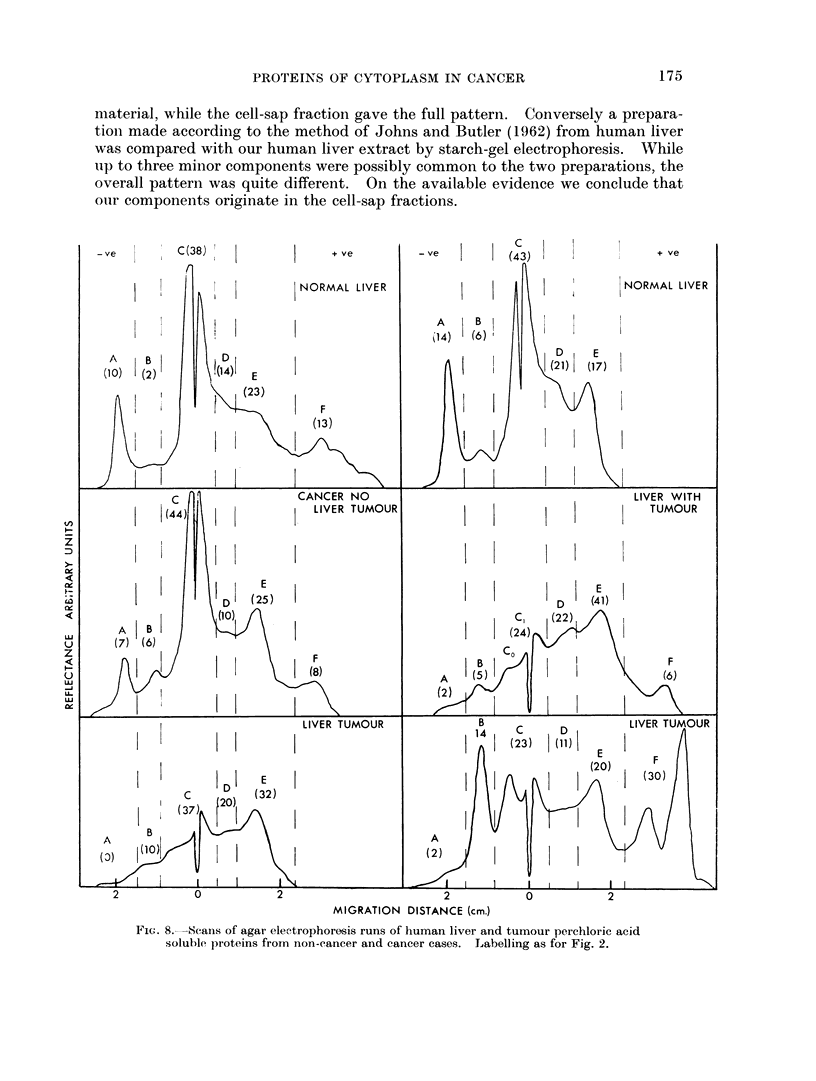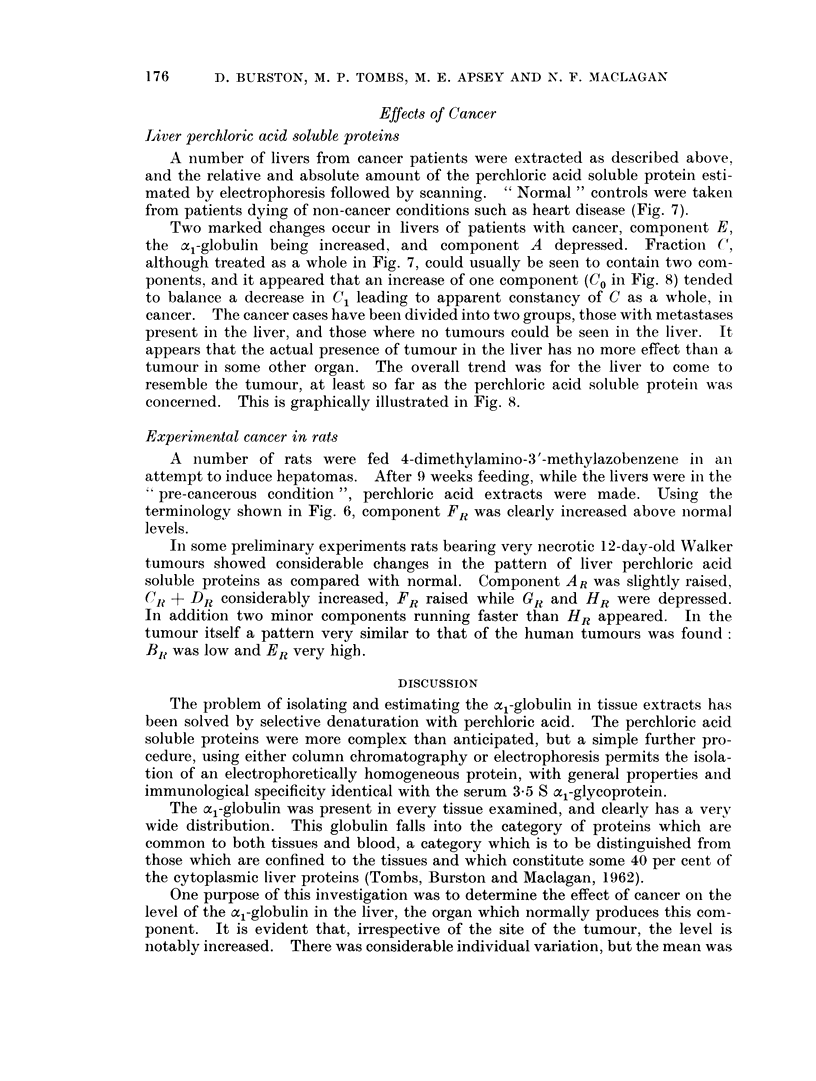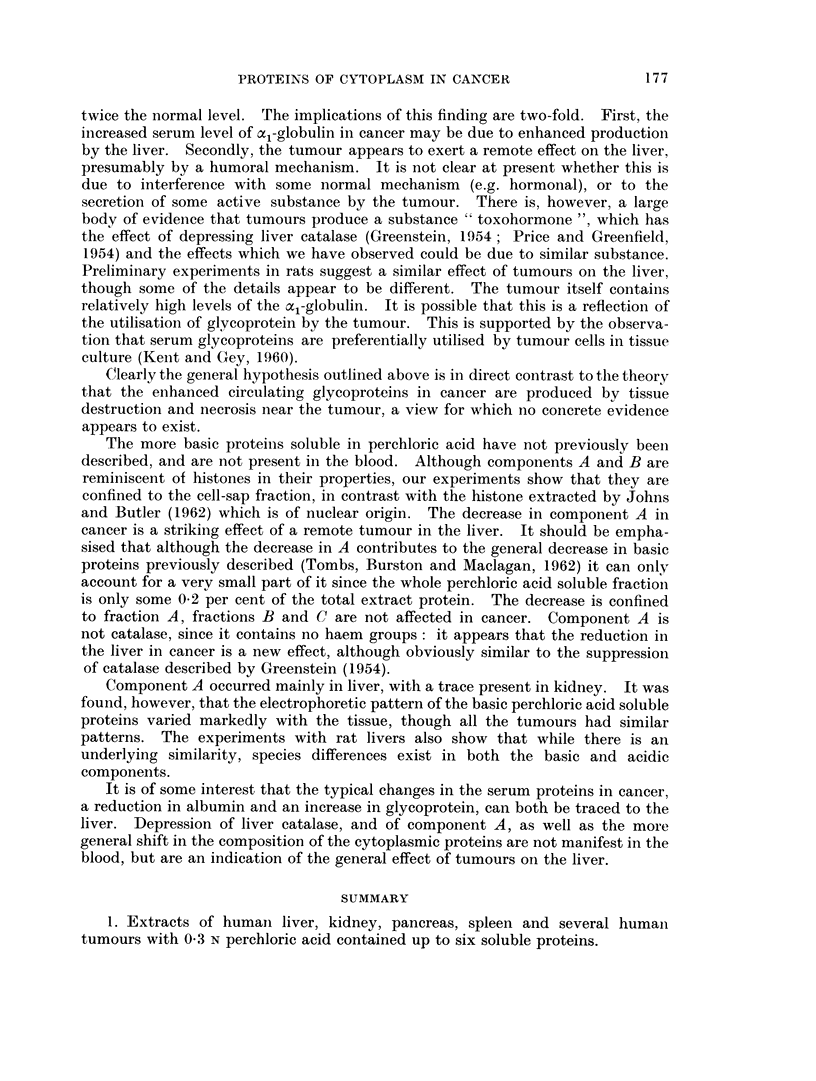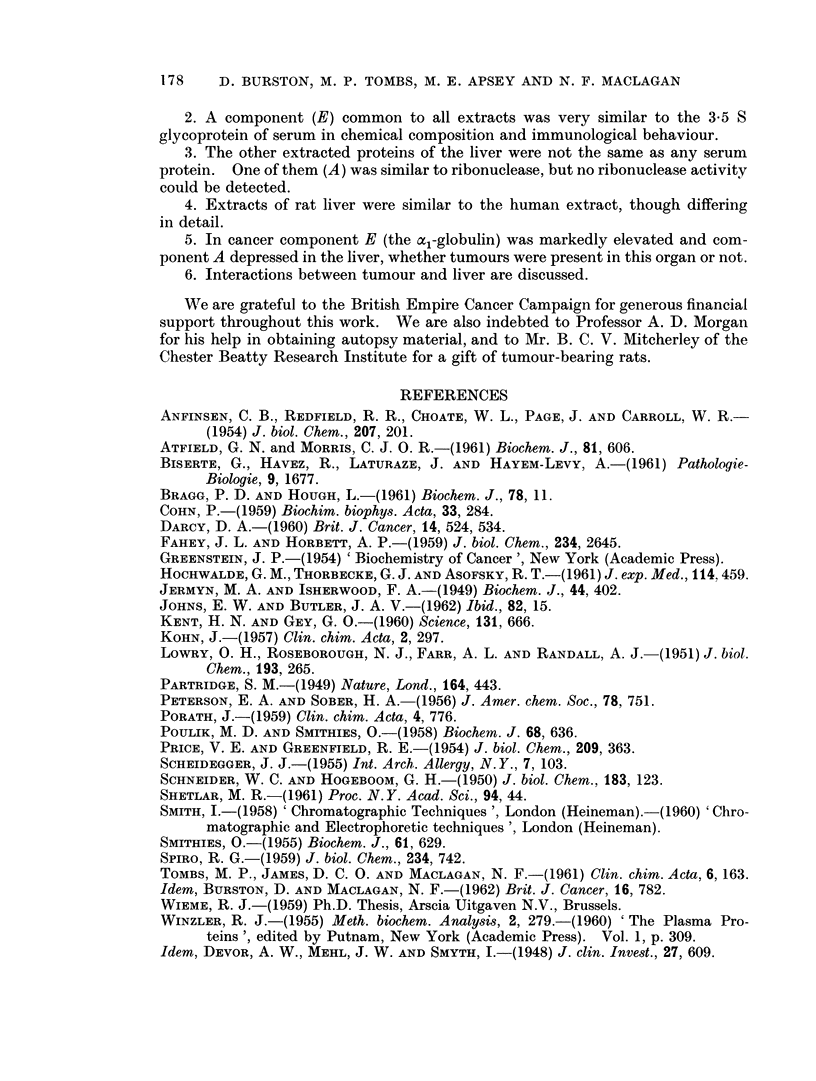# The Perchloric Acid Soluble Basic and Acidic Proteins of the Cytoplasm: Variation in Cancer

**DOI:** 10.1038/bjc.1963.24

**Published:** 1963-03

**Authors:** D. Burston, M. P. Tombs, M. E. Apsey, N. F. Maclagan


### THE PERCHLORIC ACID SOLUBLE BASIC AND ACIDIC PROTEINS OF THE CYTOPLASM: VARIATION IN CANCER

D. BURSTON, M. P. TOMBS, M. E. APSEY AND N. F. MACLAGAN

*From the Department of Chemical Pathology, Westminster Medical School, London, S.W.1*

Received for publication January 9, 1963

A CHARACTERISTIC finding in malignant disease is an increase in the circulating glycoproteins, particularly the serum $\alpha_1$-globulin. Such a change has frequently been reported in man (Winzler, 1960 ; Tombs, James and Maclagan, 1961) and in animals (Darcy, 1960). There has been some speculation as to the source and mechanism of production of the excess $\alpha_1$-glycoprotein. Suggestions have ranged from an increased production by the normal mechanism, or by injured or necrotic tissue, to a preferential utilisation of non-glycoproteins, leaving an apparent preponderance of glycoprotein in the serum (Shetlar, 1961).

Good evidence that the liver is the normal source of serum $\alpha_1$-glycoproteins has only recently become available (Spiro, 1959 ; Hochwalde, Thorbecke and Asofsky, 1961). If, in cancer, the liver is stimulated to produce more $\alpha_1$-glycoprotein, it might be expected that the organ itself would contain more than it normally does. Very little evidence is available at present on the distribution of acidic glycoproteins within normal tissue or tumours. In a previous investigation (Tombs, Burston and Maclagan, 1962) chromatographic analysis of the cytoplasmic proteins of a number of organs showed that a glycoprotein very similar to if not identical with the serum $\alpha_1$-globulin was widely distributed ; in particular it was present in the liver. Quantitative analysis of several human livers showed that in cancer the more basic and exclusively cytoplasmic proteins were depressed, and the acidic components, including the $\alpha_1$-globulin were increased. This was so whether tumours were present in the liver or not.

These results suggested that the tumour in some way caused the liver to produce more $\alpha_1$-globulin than it normally does. However, it was felt desirable to obtain more precise estimates of individual proteins than was possible from the somewhat heterogeneous chromatographic fractions. Thus in contrast with the previous experiment, which was a general analysis of all the cytoplasmic proteins, we sought a method of obtaining $\alpha_1$-globulin component in a pure state, and its relatively precise estimation.

Perchloric acid was introduced (Winzler, Devor, Mehl and Smyth, 1948) as a reagent for the selective denaturation and precipitation of all the serum proteins except a glycoprotein fraction which remains soluble. While this fraction is by no means homogeneous (Biserte, Havez, Laturaze and Hayem-Levy, 1961) the major component is the $\alpha_1$-glycoprotein. It seemed reasonable to suppose that the use of perchloric acid on tissue extracts would give good preparations of the $\alpha_1$-globulin. This was found to be so, but unexpectedly a number of basic cytoplasmic proteins were also found to be soluble in perchloric acid : these substances, one of which exhibits variation in cancer, have not previously been described.

### METHODS AND MATERIALS

### *Tissue extracts*

Tissues and organs were obtained at autopsy and either used immediately or stored at $-20°$ after a preliminary wash in cold 0·9 per cent saline.

Where possible 100 g. of tissue was taken and cut into small pieces with scissors washed with cold saline and homogenised with a volume of 0·02 M $Na_2HPO_4$ 12 $H_2O$(7·1932 g./l.) pH 8 equal to their wet weight. Homogenisation was carried out in an M.S.E. Atomix for 3 minutes. The homogenate was then centrifuged at 40,000 *g* for 30 minutes to remove particulate matter and the supernatant treated as described below. The particulate matter was discarded.

### *Perchloric acid precipitation*

(a) *Analytical.*—The volume of the tissue extract was measured and half this volume of 0·9 N perchloric acid, prepared by dilution of A.R. 60 per cent $HClO_4$ (97·2 ml./l., v/v) was added slowly with stirring. After allowing to stand for 10 minutes the solution was filtered under suction with the aid of Hyflo super-cel on No. 5 Whatman paper. The volume of the clear solution was then measured and ammonium sulphate was added to saturation (approximately 76 g./l.). After allowing to stand for 15 minutes the precipitate was sedimented at 10,000 *g* and taken up in 5–10 ml. of water. The resultant solution was then dialysed to remove ammonium sulphate and any precipitate undissolved at this stage was sedimented at 10,000 *g*. The supernatant was then freeze-dried and stored at $-20°$.

(b) *Preparative.*—This procedure was the same as the analytical except that 500–1000 g. of tissue was used and the final solution was not usually freeze-dried but was dialysed directly into the equilibrating buffer for DEAE-cellulose chromatography.

### *Protein estimation*

0·1 ml. of a solution of the extract in 0·05 M veronal buffer pH 8·6 was added to 1 ml. of distilled water and mixed. The protein concentration was then determined in two 0·1 ml. samples of this solution by the method of Lowry, Roseborough, Farr and Randall (1951), using bovine serum albumin as a standard.

### *Analytical agar electrophoresis*

This was carried out on 3 in. $\times$ 1 in. microscope slides by the method of Wieme (1959) using 1 per cent Oxoid Ionagar No. 2 in 0·025 M veronal. The electrode buffer was 0·05 M veronal pH 8·6. Electrophoresis was carried out at a current of 5 m.amp./slide until the albumin zone of a jaundiced marker serum reached a predetermined mark on the slide. The optimum conditions were determined by trial and error. After electrophoresis the slides were stained with aqueous amido-Schwartz and washed in 2 per cent acetic acid until the background was clear. After drying at room temperature, the protein pattern was scanned with an Optica reflectance Chromoscan, fitted with a red filter. From the scan, the area of the peaks was determined and thus the relative proportions of each component in the extract. The absolute amounts of each component could then be calculated from the total protein concentration of the extract and were expressed in terms of mg. protein/100 g. wet weight tissue.

*Immuno-electrophoresis*

This was carried out by a modification of the method of Scheidegger (1955) on 3 in. × 2 in. glass microscope slides. Horse anti-human serum was obtained from the Instut Pasteur, Paris and rabbit anti-rat serum from Burroughs-Welcome Ltd.

*Cellulose acetate electrophoresis*

Cellulose acetate electrophoresis was carried out by the method of Kohn (1957) on Oxoid, cellulose acetate strips, (Oxo Ltd., Southwark Bridge Road, London, S.E.1), in 0·05 M veronal pH 8·6 and a current of 2 m. amp./strip. Protein was stained with aqueous amido-Schwarz and mucopolysaccharide with alcian blue. Strips were scanned as for agar electrophoresis.

*Starch gel electrophoresis*

This was carried out by the method of Smithies (1955) in perspex trays 25 × 10 × 0·9 cm. for 18 hours at a potential of 5v/cm. length of tray.

### *Fractionation Methods*

*Cellulose ion-exchange chromatography*

(a) *DEAE-cellulose.*—This was prepared according to the method of Peterson and Sober (1956). Chromatography was carried out at room temperature on columns 13 × 2 cm., using stepwise elution or the gradient system described previously for the fractionation of tissue protein (Tombs *et al.*, 1962). In both cases the starting buffer was 0·01 M $Na_2$ $HPO_4$ 12 $H_2O$ (3·5966 g./l.) pH 8·3 and the extract was dialysed against this before application to the column. When stepwise elution was used the second buffer was 0·2 M phosphate, (0·7163$_g$ $Na_2H$ $PO_4$ 12 $H_2O$., 28·081 g. $NaH_2PO_4$ 2 $H_2O$/l., pH 5·8) and the third buffer 0·6 M $NaH_2PO_4$ 2 $H_2O$ (93·160 g./l., pH 4·3). Protein concentration in the effluent was determined on every third 6 ml. fraction collected, by the method of Lowry *et al.* (1951) as described previously. The contents of the tubes comprising each peak in the chromatogram were pooled and the first pooled fraction consisting of the basic proteins and glycogen was dialysed against 0·02 M sodium acetate adjusted to pH 4·6 with glacial acetic acid. The other pooled fractions were dialysed against water and freeze-dried.

(b) *CM-cellulose.*—Chromatography was carried out on 20 × 2 cm. columns of CM-80 cellulose (Whatman Ltd.) at room temperature and with step wise elution. Protein concentration in the effluent was not measured; every thirty factions after a change of buffer being pooled, dialysed against distilled water and freeze-dried.

The following buffers were used, in the order given :—(1) 0·02 M sodium acetate pH 4·6, (2) 0·05 M sodium acetate pH 5·0, (3) 0·07 M sodium acetate pH 5·5, (4) 0·02 M sodium acetate pH 6·0. All were adjusted to the required pH with glacial acetic acid.

*Preparative electrophoresis*

Preparative electrophoresis was carried out by the method of Fahey and Horbett (1959) on polyvinyl chloride (Geon 425) blocks. Geon 425 was obtained from British Geon Ltd. and after washing in glacial acetic acid followed by distilled water until neutral, the resulting slurry was dried under vacuum, keeping the temperature below 37°.

The dried Geon powder was then suspended in 0·05 M veronal buffer 8·6 to form a slurry and was poured into a perspex tray of dimensions 25 × 5 × 0·9 cm. Excess buffer was removed by blotting with filter paper. Wicks consisting of three layers of Whatman No. 3 MM filter paper the width of the tray, connected the ends of the tray to the buffer vessels. These were large perspex tanks containing 0·05 M veronal pH 8·6.

70·4 mg. of the freeze-dried solid was dissolved in 0·4 ml. of veronal, giving a turbid solution. This was applied in a slot 3 × 0·2 × 0·9 cm., 10 cm. from the end of the tray connected to the positive electrode. When the sample had soaked into the Geon the edges of the slot were pressed together. Electrophoresis was then carried out at a current of 20 m. amp. with cooling to 4° for 2 hours and for a further 16 hours at 5 m. amp. with no cooling. At the end of this time the block was cut into centimetre sections and the protein displaced with 3 ml. of 0·9 per cent sodium chloride, filtering under vacuum through a sintered funnel. The resultant solutions were all brought to a volume of 5 ml. with distilled water. Protein estimations were then made by the method of Lowry *et al.* (1951) and the diagram in Fig. 4 plotted. Sections of the block representing protein peaks were pooled and freeze-dried after dialysis against distilled water. The composition of the freeze-dried material was checked by agar electrophoresis.

*Gel filtration*

Fractionation employing gel filtration was carried out on columns, 48 × 2 cm. diameter of Sephadex G 200 (Porath, 1959) equilibrated with water. Freeze-dried solid (262 mg.) containing about 30 mg. of protein (the remainder being glycogen) was applied to the column in 5 ml. of water. The column was developed with distilled water, the whole experiment being at room temperature (18°). Fractions of 3 ml. volume were collected. Protein estimations were carried out by the method of Lowry *et al.* (1951) on every second fraction. Glycogen was detected by measurement of the turbidity at 650 m$\mu$. Fractions corresponding to a single peak were then pooled and freeze-dried : the composition of this was then examined by agar electrophoresis.

*Organic solvent fractionation*

(1) *Acetone.*—In an attempt to speed up the preparative procedure, acetone fractionation of the filtrate after perchloric acid precipitation was attempted. The acetone was added slowly at 4° with stirring. After standing for 15 minutes the precipitate was centrifuged down at 10,000 **g**, washed with appropriate acetone water mixtures, recentrifuged, and taken up in distilled water and freeze-dried ; however a large part of the precipitate failed to redissolve. More acetone was added to the supernatant to give final concentrations of 10, 20, 30 and 60 per cent (v/v). After the 60 per cent precipitate had been centrifuged down the supernatant solution was saturated with ammonium sulphate and again centrifuged at 10,000 **g**. The precipitate was taken up in distilled water, dialysed and freeze-dried. The composition of the resultant freeze-dried solids was determined by agar electrophoresis.

(2) *Methanol.*—This fractionation was carried out on a freeze-dried perchloric acid extract of liver. This was dissolved in water, clarified by centrifugation, and methanol added as for acetone (above) to give final concentrations of 10, 20, 30, 40 and 60 per cent (v/v), allowing the mixture to stand for 15 minutes after each addition. Any precipitate formed was centrifuged down at 10,000 g and dissolved in 0·5 ml. of distilled water and freeze-dried. The supernatant from the 60 per cent methanol precipitation was also freeze-dried.

*Ultraviolet Absorption Spectra*

A weighed amount of the freeze-dried solid was dissolved in 3 ml. of distilled water to give an approximately 0·1 per cent solution. Any undissolved material present at this stage was centrifuged down and the concentration of protein in the solution was determined by dry weight at the end of the experiment (1 ml. samples were evaporated to dryness at 105° over-night). Spectra were measured at intervals of 2 m$\mu$. in cuvettes of 1 cm. path length against distilled water.

*Carbohydrate Determinations*

(1) *Qualitative*

Freeze-dried material (5–10 mg.) was dissolved in 3 ml. of water and hydrolysed for 24 hours by the ion-exchange method of Bragg and Hough (1961). At the end of this time the resin was filtered off and the filtrate taken down to dryness in a vacuum oven. The residue was dissolved in 0·2 ml. of water and examined by paper chromatography, using the hanging strip method of Jermyn and Isherwood (1949), and ethyl acetate, pyridine, water (12 : 5 : 4) as solvent. Sugars were detected by staining with aniline hydrogen phthalate (Partridge, 1949).

(2) *Quantitative*

*Hexose.*—This was determined by the orcinol method described by Winzler (1955) on solutions of the freeze-dried solids (approximately 1 mg./ml.) Galactose and mannose in a ratio of 1 : 1 were used as a standard.

*Hexosamine.*—This was determined by the Elson-Morgan method as described by Winzler (1955) on the same solutions as above. Glucosamine was used as a standard.

*Ribonuclease Determinations*

Ribonuclease determinations were made by the method of Anfinsen, Redfield, Choate, Page and Carroll (1954) on whole perchloric acid extracts of various tissues. Ribonuclease (40 Kunitz units/mg. L. Light and Co. Ltd.), was use to construct a calibration curve using dialysed RNA (Nutritional Biochemical Corporation). Determinations were carried out on 0·05 ml. of whole perchloric extract corresponding to about 1 mg. of protein.

*Amino Acid Compositions*

This was investigated using the method described by Atfield and Morris (1961). Freeze-dried material (5–6 mg.) was dissolved in 2 ml. of 6 N HCl and heated in a boiling water bath for 24 hours. The hydrolysates were then prepared as described by Atfield and Morris (1961), and examined in a Shandon high voltage electrophoresis apparatus.

After electrophoresis amino acids were detected with ninhydrin, Pauli reagent (for histidine) and Sakaguchi reagent (for arginine) according to Smith (1958).

*Rat Liver Extracts*

Livers were removed as quickly as possible after the animals (Wistar strain) had been killed with ether and bled by heart puncture. The livers were washed with 0·9 per cent saline and used immediately or stored at $-20°$. Each liver was homogenised for $1\frac{1}{2}$ minutes in nine volumes of the following medium :—(Cohn, 1959) 0·25 M sucrose, 0·025 M KCl, 0·035 M $KHCO_3$, 0·004 M $MgCl_2$, 0·008 M $K_2HPO_4$ and 0·0013 M $KH_2PO_4$. The homogenate was centrifuged at 5000 *g* for 10 minutes and the supernatant centrifuged at 57,000 *g* for 60 minutes to sediment the microsomes. The supernatant fraction was then treated with perchloric acid as for human tissue.

*Cell Fractionation*

Cell fractionation of a rat liver was carried out by the method of Schneider and Hogeboom (1950) using the medium of Cohn (1959) (see above) instead of 0·25 M sucrose. After homogenisation in 9 volumes of the medium for $1\frac{1}{2}$ minutes in an M.S.E. Atomix the homogenate was centrifuged as usual, to give nuclei (sedimented at 700 *g*) mitochondria (24,000 *g*) microsomes (105,000 *g*) and supernatant fractions. All the sediments were washed once by resuspension and recentrifuged. Suspensions of each fraction were then extracted with perchloric acid as above.

### RESULTS

Attempts to isolate $\alpha_1$-globulin-like components from tissues led us to consider the method of differential denaturation with perchloric acid used on plasma proteins by Winzler *et al.* (1948). Fig. 1 shows the total amount of protein unprecipitated at various strengths of perchloric acid in plasma and a liver extract containing a comparable amount of total protein. The plasma results were similar to those described by Winzler *et al.* (1948). The liver extract exhibited a somewhat different behaviour, since a maximum solubility was found at approximately 0·3 N perchloric acid. As this was also the lowest concentration at which all albumin was reliably precipitated, it was chosen for routine use.

The electrophoretic properties of the plasma extract (Fig. 2) were in close agreement with Winzler (1948), consisting predominantly of $\alpha_1$-globulin. The liver extracts contained, unexpectedly, a number of other components (*A* to *F*) ; the $\alpha_1$-globulin (*E*), though always present was by no means the predominating component. Different tissues contained quite different amounts of the more basic proteins (*A*, *B* and *C* in Fig. 2). The liver contained the highest proportion, particularly of A, of any tissue examined.

Three methods were explored for further fractionation of the liver extract. The position was complicated by the presence in some livers of large amounts of glycogen, which appeared in the final perchloric acid soluble fraction. A complete fractionation scheme, using alternating chromatography on DEAE and CM-cellulose is illustrated in Fig. 3. It was comparatively simple to separate the $\alpha_1$-globulin, but fractionation of the basic components was more difficult. Component *A* eluted anomalously early. A further difficulty was the considerable individual variation encountered from one liver to another, tending to upset fixed elution schedules. Nevertheless substantial purification of the components was achieved.

Large scale electrophoresis on Geon resin was quite feasible and gave a useful separation (Fig. 4). Gel filtration on Sephadex gave some fractionation—the

$\alpha_1$-globulin was obtained with $\alpha_2$-globulin contamination ; the main interest in this procedure is, however, the information it yields on relative molecular sizes (see below).

A few experiments were tried using organic solvents on the initial perchloric acid extract. These were not promising, though 60 per cent acetone (v/v) precipitated all except component A from liver extracts, offering an easy approach to this substance. Generally precipitates from acetone or methanol could not be redissolved : the part that could be redissolved appeared to be mainly component C.

FIG. 1.—Total protein soluble in various concentrations of perchloric acid, from 90 ml. of plasma (approximately 4 g. protein, shown by ●) and from 100 ml. of liver extract (approximately 4 g. protein, shown by ○).

*Properties of perchloric acid soluble protein*

The following comments apply to human liver perchloric acid soluble protein, and were measured mainly on components purified by the chromatographic method (Fig. 3).

*Component F.*—This fraction contained at least two mucopolysaccharide components staining with alcian blue after electrophoresis.

*Component E.*—This fraction was of $\alpha_1$-globulin mobility and immunoelectrophoresis experiments both directly and with adsorbed anti-sera showed the major component to be immunologically identical with the 3·5 S $\alpha_1$-glycoprotein of serum. Other components were shown by starch-gel electrophoresis. The major component migrated as a post-albumin (3·5 S glycoprotein). Traces of a component with $\alpha_1$ B globulin mobility (Poulik and Smithies, 1958) and of orosomucoid were also present. The perchloric acid filtrates from plasma, in contrast, contained a much higher proportion (about 70 per cent) of orosomucoid. The

FIG. 2.—Scans of agar-gel electrophoresis runs in veronal buffer pH 8·6 of perchloric acid extracts of various human tissues. A whole serum from an advanced cancer case is shown for comparison. The figures in parentheses next to the letters labelling components are the relative proportions (per cent) of each component.

fraction contained appreciable amounts of carbohydrate : the periodic-acid-Schiff reaction applied to electrophoresis strips (Smith, 1958) was strongly positive, and after hydrolysis of the isolated solid (from chromatography, see above) paper chromatography revealed galactose and mannose in roughly equal proportions. Two preparations gave analyses as follows : (1) hexose 6·3 per cent, hexosamine 4·0 per cent, $N$ (Kjeldahl) 10·3 per cent, protein by biuret 65 per cent. (2) hexose 6·1 per cent, hexosamine 3·1 per cent, $N$ (Kjeldahl) 8·2 per cent, protein by biuret 65 per cent.

The absorption spectrum (Fig. 5) showed a maximum at 277 m$\mu$. and $E_{1cm.}$ 280 m$\mu$. 1 per cent = 4·6. The gel filtration behaviour (Fig. 4) was consistent with a molecular weight of roughly 60,000. The properties are all consistent with the reported composition of the 3·5 S $\alpha_1$-glycoprotein of serum (Winzler, 1960) as the major component of fraction *E*.

*Component D.*—This does not appear to be a serum component by the immuno-electrophoretic criterion, although it bears a superficial similarity to serum

FIG. 3.—Diagram showing the procedure for chromatographic fractionation of human liver perchloric acid soluble proteins. The whole extract was applied first to DEAE-cellulose, and the part not bound to DEAE transferred to CM-cellulose. Bound components were eluted with the buffers shown. At the right is shown, diagrammatically, the result of agar electrophoresis of each fraction, after concentration.

$\alpha_2$-globulins. It contains carbohydrate by the periodic-acid-Schiff reaction and from its behaviour on Sephadex G200 (Fig. 4) its molecular weight is greater than 60,000.

*Component C.*—Immuno-electrophoresis indicated that there were only traces of serum components present in this fraction.

Ion-exchange hydrolysis followed by paper chromatography showed the presence of glucose, 6 per cent in one preparation and 4 per cent in another. As this component is eluted immediately after glycogen from the CM-cellulose column (see Fig. 3) it may represent glycogen trail. The absence of hexosamine in this fraction also suggests that it is not a glycoprotein.

The ultraviolet absorption curve (Fig. 5) was not that of a typical protein. Although there was a maximum in the region of 280 mμ., the region 280–250 mμ. was fairly flat with several minor peaks in the region 270–260 mμ. and with only a shallow dip in the region 250–240 mμ. In addition the component was yellowish in solution, suggesting the presence of bound pigments.

FIG. 4.—Fractionation of human liver perchloric acid soluble protein. At the top is shown the result of a large scale preparative electrophoresis experiment on Geon resin in veronal pH 8·6 buffer.

At the bottom the result of filtration on Sephadex G 200, in water. To the right is shown, diagrammatically, agar electrophoresis of the resulting fractions, after dialysis and concentration. For further details see text.

Amino acid analyses indicated that lysine predominated in the basic amino acids. Arginine was also present but only a trace of histidine.

Gel filtration suggested that the molecular weight of component *C* is somewhat higher than that of ribonuclease, used as a test substance and is probably about 20,000–30,000.

*Component B*.—This is not a serum component as judged by immuno-electrophoresis against an anti-human serum. Quantitative estimation indicated a hexose level of 1 per cent but this was all glucose and probably represents glycogen contamination.

FIG. 5.—Ultraviolet absorption spectra of human liver perchloric acid soluble protein in water. The results have been calculated to 1 per cent protein in each case.

The ultraviolet absorption curve was similar to that of component *C* with a maximum in the region of 280 mμ. and a flat region with some minor peaks in the region 280–250 mμ. The low specific extinction $E^{1\%}_{1 cm.}$ 280 mμ. = 3·0 suggests a low aromatic amino acid content.

Amino acid analysis indicated that in the basic amino acids lysine predominated with some arginine and only a trace or no histidine.

*Component A*.—This protein was also absent from serum as judged by immuno-electrophoresis. There was no hexose other than a trace of glucose from " glycogen trail ". It dissolved in water to give a faintly yellow solution but did not contain any haem (as tested by the benzidine reaction). The ultraviolet absorption curve (Fig. 5) is that of a typical protein with a maximum at 278 mμ. and a shoulder at 278–285 mμ.

A highly purified sample of *A* had a specific extinction of 5 at 280 mμ. indicating a higher aromatic amino acid content than the other basic proteins described (*B* and *C*). The shape of the absorption curve suggests that the major aromatic amino acid present is tyrosine with little or no tryptophan. Gel filtration experi. ments (Fig. 4) suggest a molecular weight similar to that of ribonuclease (14,000).

It is a very stable protein and is not precipitated from its perchloric acid solution by organic solvents. Histidine was particularly abundant, though lysine and arginine were also present.

The molecular weight, ultraviolet absorption curve and to some extent the basic acid composition were similar to those of ribonuclease. However ribonuclease activity was absent.

*Rat perchloric acid soluble proteins*

A comparison of rat serum, perchloric acid extract of serum, liver cytoplasmic protein and its perchloric acid extract is shown in Fig. 6. It is clear that, although

FIG. 6.—Scans of cellulose acetate electrophoresis runs of rat serum, its perchloric acid extract, rat liver cell-sap protein and its corresponding perchloric acid extract. The relative proportions of each electrophoretic fraction are shown in parentheses.

there are differences of detail between human and rat extract, the overall pattern of basic and acidic protein is very similar.

Experiments similar to those described above for human extracts, using an anti-rat serum protein antiserum, showed that components, $E_R$ and $F_R$ (Fig. 6) contained serum proteins, but the remainder appear to be confined to the tissue.

Rats were used to investigate the effect of blood contamination : the livers of six rats perfused with saline before extraction, were compared with six unperfused livers. Perfusion had no significant effect on the level of any component and calculations based on blood volume in the liver, and the level of various blood proteins also suggest that blood contamination is unimportant.

FIG. 7.—Distributions of human liver perchloric acid soluble proteins in : ○, non-cancer liver ; ●, cancer cases, but no liver tumours ; ◑, cancer cases with liver metastases present. Some typical tumour results are also shown (X).

*Cytoplasmic origin of perchloric acid soluble protein*

Although the general procedure used for the human tissue extract would be expected to leave only the cell-sap fraction in solution, the report by Johns and Butler (1962) that some nuclear components can be extracted by 0·75 N $HClO_4$ led us to investigate this point further. Using rat liver, a complete cell fractionation was carried out in a sucrose medium into nuclei, mitochondria, microsomes and cell-sap fraction. Each was then extracted according to our usual procedure : the nuclei and mitochondria yielded nothing, the microsomes a trace of very basic material, while the cell-sap fraction gave the full pattern. Conversely a preparation made according to the method of Johns and Butler (1962) from human liver was compared with our human liver extract by starch-gel electrophoresis. While up to three minor components were possibly common to the two preparations, the overall pattern was quite different. On the available evidence we conclude that our components originate in the cell-sap fractions.

FIG. 8.—Scans of agar electrophoresis runs of human liver and tumour perchloric acid soluble proteins from non-cancer and cancer cases. Labelling as for Fig. 2.

### *Effects of Cancer*

*Liver perchloric acid soluble proteins*

A number of livers from cancer patients were extracted as described above, and the relative and absolute amount of the perchloric acid soluble protein estimated by electrophoresis followed by scanning. " Normal " controls were taken from patients dying of non-cancer conditions such as heart disease (Fig. 7).

Two marked changes occur in livers of patients with cancer, component $E$, the $\alpha_1$-globulin being increased, and component $A$ depressed. Fraction $C$, although treated as a whole in Fig. 7, could usually be seen to contain two components, and it appeared that an increase of one component ($C_0$ in Fig. 8) tended to balance a decrease in $C_1$ leading to apparent constancy of $C$ as a whole, in cancer. The cancer cases have been divided into two groups, those with metastases present in the liver, and those where no tumours could be seen in the liver. It appears that the actual presence of tumour in the liver has no more effect than a tumour in some other organ. The overall trend was for the liver to come to resemble the tumour, at least so far as the perchloric acid soluble protein was concerned. This is graphically illustrated in Fig. 8.

*Experimental cancer in rats*

A number of rats were fed 4-dimethylamino-3′-methylazobenzene in an attempt to induce hepatomas. After 9 weeks feeding, while the livers were in the " pre-cancerous condition ", perchloric acid extracts were made. Using the terminology shown in Fig. 6, component $F_R$ was clearly increased above normal levels.

In some preliminary experiments rats bearing very necrotic 12-day-old Walker tumours showed considerable changes in the pattern of liver perchloric acid soluble proteins as compared with normal. Component $A_R$ was slightly raised, $C_R + D_R$ considerably increased, $F_R$ raised while $G_R$ and $H_R$ were depressed. In addition two minor components running faster than $H_R$ appeared. In the tumour itself a pattern very similar to that of the human tumours was found : $B_R$ was low and $E_R$ very high.

### DISCUSSION

The problem of isolating and estimating the $\alpha_1$-globulin in tissue extracts has been solved by selective denaturation with perchloric acid. The perchloric acid soluble proteins were more complex than anticipated, but a simple further procedure, using either column chromatography or electrophoresis permits the isolation of an electrophoretically homogeneous protein, with general properties and immunological specificity identical with the serum 3·5 S $\alpha_1$-glycoprotein.

The $\alpha_1$-globulin was present in every tissue examined, and clearly has a very wide distribution. This globulin falls into the category of proteins which are common to both tissues and blood, a category which is to be distinguished from those which are confined to the tissues and which constitute some 40 per cent of the cytoplasmic liver proteins (Tombs, Burston and Maclagan, 1962).

One purpose of this investigation was to determine the effect of cancer on the level of the $\alpha_1$-globulin in the liver, the organ which normally produces this component. It is evident that, irrespective of the site of the tumour, the level is notably increased. There was considerable individual variation, but the mean was twice the normal level. The implications of this finding are two-fold. First, the increased serum level of $\alpha_1$-globulin in cancer may be due to enhanced production by the liver. Secondly, the tumour appears to exert a remote effect on the liver, presumably by a humoral mechanism. It is not clear at present whether this is due to interference with some normal mechanism (e.g. hormonal), or to the secretion of some active substance by the tumour. There is, however, a large body of evidence that tumours produce a substance " toxohormone ", which has the effect of depressing liver catalase (Greenstein, 1954; Price and Greenfield, 1954) and the effects which we have observed could be due to similar substance. Preliminary experiments in rats suggest a similar effect of tumours on the liver, though some of the details appear to be different. The tumour itself contains relatively high levels of the $\alpha_1$-globulin. It is possible that this is a reflection of the utilisation of glycoprotein by the tumour. This is supported by the observation that serum glycoproteins are preferentially utilised by tumour cells in tissue culture (Kent and Gey, 1960).

Clearly the general hypothesis outlined above is in direct contrast to the theory that the enhanced circulating glycoproteins in cancer are produced by tissue destruction and necrosis near the tumour, a view for which no concrete evidence appears to exist.

The more basic proteins soluble in perchloric acid have not previously been described, and are not present in the blood. Although components *A* and *B* are reminiscent of histones in their properties, our experiments show that they are confined to the cell-sap fraction, in contrast with the histone extracted by Johns and Butler (1962) which is of nuclear origin. The decrease in component *A* in cancer is a striking effect of a remote tumour in the liver. It should be emphasised that although the decrease in *A* contributes to the general decrease in basic proteins previously described (Tombs, Burston and Maclagan, 1962) it can only account for a very small part of it since the whole perchloric acid soluble fraction is only some 0·2 per cent of the total extract protein. The decrease is confined to fraction *A*, fractions *B* and *C* are not affected in cancer. Component *A* is not catalase, since it contains no haem groups: it appears that the reduction in the liver in cancer is a new effect, although obviously similar to the suppression of catalase described by Greenstein (1954).

Component *A* occurred mainly in liver, with a trace present in kidney. It was found, however, that the electrophoretic pattern of the basic perchloric acid soluble proteins varied markedly with the tissue, though all the tumours had similar patterns. The experiments with rat livers also show that while there is an underlying similarity, species differences exist in both the basic and acidic components.

It is of some interest that the typical changes in the serum proteins in cancer, a reduction in albumin and an increase in glycoprotein, can both be traced to the liver. Depression of liver catalase, and of component *A*, as well as the more general shift in the composition of the cytoplasmic proteins are not manifest in the blood, but are an indication of the general effect of tumours on the liver.

### SUMMARY

1. Extracts of human liver, kidney, pancreas, spleen and several human tumours with 0·3 N perchloric acid contained up to six soluble proteins.

2. A component ($E$) common to all extracts was very similar to the 3·5 S glycoprotein of serum in chemical composition and immunological behaviour.

3. The other extracted proteins of the liver were not the same as any serum protein. One of them ($A$) was similar to ribonuclease, but no ribonuclease activity could be detected.

4. Extracts of rat liver were similar to the human extract, though differing in detail.

5. In cancer component $E$ (the $\alpha_1$-globulin) was markedly elevated and component $A$ depressed in the liver, whether tumours were present in this organ or not.

6. Interactions between tumour and liver are discussed.

We are grateful to the British Empire Cancer Campaign for generous financial support throughout this work. We are also indebted to Professor A. D. Morgan for his help in obtaining autopsy material, and to Mr. B. C. V. Mitcherley of the Chester Beatty Research Institute for a gift of tumour-bearing rats.

### REFERENCES

ANFINSEN, C. B., REDFIELD, R. R., CHOATE, W. L., PAGE, J. AND CARROLL, W. R.—(1954) *J. biol. Chem.*, **207**, 201.
ATFIELD, G. N. and MORRIS, C. J. O. R.—(1961) *Biochem. J.*, **81**, 606.
BISERTE, G., HAVEZ, R., LATURAZE, J. AND HAYEM-LEVY, A.—(1961) *Pathologie-Biologie*, **9**, 1677.
BRAGG, P. D. AND HOUGH, L.—(1961) *Biochem. J.*, **78**, 11.
COHN, P.—(1959) *Biochim. biophys. Acta*, **33**, 284.
DARCY, D. A.—(1960) *Brit. J. Cancer*, **14**, 524, 534.
FAHEY, J. L. AND HORBETT, A. P.—(1959) *J. biol. Chem.*, **234**, 2645.
GREENSTEIN, J. P.—(1954) ' Biochemistry of Cancer ', New York (Academic Press).
HOCHWALDE, G. M., THORBECKE, G. J. AND ASOFSKY, R. T.—(1961) *J. exp. Med.*, **114**, 459.
JERMYN, M. A. AND ISHERWOOD, F. A.—(1949) *Biochem. J.*, **44**, 402.
JOHNS, E. W. AND BUTLER, J. A. V.—(1962) *Ibid.*, **82**, 15.
KENT, H. N. AND GEY, G. O.—(1960) *Science*, **131**, 666.
KOHN, J.—(1957) *Clin. chim. Acta*, **2**, 297.
LOWRY, O. H., ROSEBOROUGH, N. J., FARR, A. L. AND RANDALL, A. J.—(1951) *J. biol. Chem.*, **193**, 265.
PARTRIDGE, S. M.—(1949) *Nature, Lond.*, **164**, 443.
PETERSON, E. A. AND SOBER, H. A.—(1956) *J. Amer. chem. Soc.*, **78**, 751.
PORATH, J.—(1959) *Clin. chim. Acta*, **4**, 776.
POULIK, M. D. AND SMITHIES, O.—(1958) *Biochem. J.* **68**, 636.
PRICE, V. E. AND GREENFIELD, R. E.—(1954) *J. biol. Chem.*, **209**, 363.
SCHEIDEGGER, J. J.—(1955) *Int. Arch. Allergy, N.Y.*, **7**, 103.
SCHNEIDER, W. C. AND HOGEBOOM, G. H.—(1950) *J. biol. Chem.*, **183**, 123.
SHETLAR, M. R.—(1961) *Proc. N.Y. Acad. Sci.*, **94**, 44.
SMITH, I.—(1958) ' Chromatographic Techniques ', London (Heineman).—(1960) ' Chromatographic and Electrophoretic techniques ', London (Heineman).
SMITHIES, O.—(1955) *Biochem. J.*, **61**, 629.
SPIRO, R. G.—(1959) *J. biol. Chem.*, **234**, 742.
TOMBS, M. P., JAMES, D. C. O. AND MACLAGAN, N. F.—(1961) *Clin. chim. Acta*, **6**, 163.
*Idem*, BURSTON, D. AND MACLAGAN, N. F.—(1962) *Brit. J. Cancer*, **16**, 782.
WIEME, R. J.—(1959) Ph.D. Thesis, Arscia Uitgaven N.V., Brussels.
WINZLER, R. J.—(1955) *Meth. biochem. Analysis*, **2**, 279.—(1960) ' The Plasma Proteins ', edited by Putnam, New York (Academic Press). Vol. 1, p. 309.
*Idem*, DEVOR, A. W., MEHL, J. W. AND SMYTH, I.—(1948) *J. clin. Invest.*, **27**, 609.